# Convergent evolution of intestinal lineages in the phylum *Methanobacteriota*

**DOI:** 10.1186/s40168-026-02434-5

**Published:** 2026-05-02

**Authors:** Evgenii Protasov, Undine S. Mies, Cathrin Spröer, Boyke Bunk, Sebastian Cristian Treitli, Katja Platt, Andreas Brune

**Affiliations:** 1 Research Group Insect Gut Microbiology and Symbiosis, Max Planck Institute for Terrestrial Microbiology, Marburg, Germany; 2 Department of Bioinformatics, Leibniz Institute DSMZ-German Collection of Microorganisms and Cell Cultures, Braunschweig, Germany

**Keywords:** Methanogens, Evolution, Gut microbiota, Archaea, Taxonomy, *Methanobacteriales*, *Methanobrevibacter*, *Methanosphaera*

## Abstract

**Background:**

Representatives of the phylum *Methanobacteriota* occur in various anoxic environments, but only members of the genera *Methanosphaera* and *Methanobrevibacter* exclusively colonize the digestive tract of animals. Recent phylogenomic analyses revealed that the genus *Methanobrevibacter*, which harbors the majority of the intestinal species, is severely underclassified and represents a family-level taxon, “*Methanobrevibacteraceae*”, that evolved entirely in the digestive tract of animals.

**Results:**

Comparative genome analysis of 158 species of *Methanobacteriota*, including uncultured representatives in the Genome Taxonomy Database (GTDB), demonstrated that the intestinal lineages are clearly separated from the remaining members of the phylum. They differ from the non-intestinal lineages in genome size, GC content, coding density, an increased number of pseudogenes and adhesin-like proteins, and show numerous adaptations to the copiotrophic gut environment. A decreased biosynthetic potential led to a dependence on other community members and limits the dispersal of intestinal species into other habitats, which is reflected in coevolutionary patterns with their major host groups among arthropods, ungulates, and primates. Certain lineages even engaged in symbiotic associations with intestinal protists, presumably benefiting from the H_2_ produced by the hydrogenosomes of their anaerobic hosts.

**Conclusions:**

Our results reveal that the transition of free-living *Methanobacteriota* to a host-associated lifestyle involves the same genomic changes that were previously recognized in gut bacteria and bacterial endosymbionts of protists, reflecting resemblances between the two prokaryotic domains that are caused by evolutionary convergence in similar environments.

**Supplementary Information:**

The online version contains supplementary material available at 10.1186/s40168-026-02434-5.

## Introduction

Members of the phylum *Methanobacteriota* occur in a wide range of anoxic environments, including marine and freshwater sediments and the deep biosphere. However, only two clades consistently colonize the digestive tract of animals. They are represented by the genera *Methanobrevibacter*, whose members are found in both invertebrates and vertebrate hosts, and *Methanosphaera*, whose members occur exclusively in mammals [1–4]. Both 16S rRNA and multi-locus gene sequence analyses of the genus *Methanobrevibacter* indicated the presence of several subclades that apparently coevolved with different host groups (i.e., ruminants, humans, and termites) [5, 6].

A comprehensive phylogenomic analysis of all archaeal isolates and metagenome-assembled genomes (MAGs) in the Genome Taxonomy Database (GTDB) of *Methanobacteriota* corroborated this notion and suggested that the high relative evolutionary divergence (RED) within the genus *Methanobrevibacter* requires the introduction of four additional genus-level taxa [7]. After inclusion of numerous MAGs from invertebrate hosts, Protasov et al. (2023) proposed a taxonomic revision of the *Methanobrevibacter* supergroup into nine genera, which included the proposal of eight new genera and the reclassification of twelve species. The names were validly published under the *Code of Nomenclature of Prokaryotes Described from Sequence Data* (SeqCode; [8]) based on genomes as type material. The validation of the new taxa and new combinations under the rules of the *International Code of Nomenclature of Prokaryotes* (ICNP), including their reclassification in the new family, “*Methanobrevibacteraceae*”, is in preparation.

Here, we provide a comparative genome analysis of the phylum *Methanobacteriota*, focusing on functional adaptations associated with the transition from a free-living to an intestinal lifestyle. It is well established that the transition of bacteria from a free-living to a host-associated lifestyle is associated with fundamental changes in their genomes [9]. However, there are only a few studies on this topic in archaea. They concern intestinal lineages among *Methanomassiliicoccales* [10], *Methanosarcinales* [11, 12], and *Methanobacteriales*. The latter have been studied in more detail [6, 13, 14], but a comprehensive analysis of *Methanobacteriota* and the evolutionary patterns associated with the transition to the copiotrophic intestinal environment is so far lacking. We investigated genome erosion, the concomitant loss of biosynthetic capacities, and the convergent gain of metabolic traits in the intestinal lineages, and aligned them with environmental factors such as physicochemical environment, host diet and ecosystem services provided by other microbiota.

## Methods

### Genome sequencing and annotation

Cultures of *M. acididurans* (DSM 15163) and *M. shimae* (DSM 116169) were obtained from Deutsche Sammlung für Mikroorganismen und Zellkulturen, Braunschweig, Germany, and DNA was extracted as previously described [2]. SMRTbell® template libraries were prepared according to the instructions from PacificBiosciences, Menlo Park, CA, USA, following the Procedure & Checklist–Preparing whole genome and metagenome libraries using SMRTbell® prep kit 3.0. Briefly, for preparation of 10-kb libraries, 2 µg genomic DNA was sheared using a Megaruptor® 3 (Diagenode, Denville, NJ, USA) according to the manufacturer’s instructions. DNA was end-repaired and ligated to barcoded adapters applying components from the SMRTbell® prep kit 3.0 (Pacific Biosciences). Reactions were carried out according to the manufacturer’s instructions. Samples were pooled equimolar. Conditions for annealing of sequencing primers and binding of polymerase to purified SMRTbell® template were assessed according to the Sample Setup in SMRT®link (PacificBiosciences). Libraries were sequenced on a SequelIIe (PacificBiosciences), taking one 30-h movie per SMRT cell.

Long-read genome assembly was performed with the “Microbial Genome Analysis” protocol included in SMRTlink version 13.0 using a target genome size of 5 Mbp. One circular chromosomal contig was obtained and rotated to the *cdc6* gene. Genomes were automatically annotated at NCBI using the PGAP pipeline.

### Phylogenomic analysis

Genomes were classified within the taxonomic framework of the Genome Taxonomy Database (GTDB, release 220) using the GTDB toolkit (v. 2.4.0) [15]. A maximum-likelihood tree was inferred from a concatenated alignment of 53 archaeal single-copy marker genes generated with the GTDB toolkit, using the IQ-Tree 2 tool with LG + F + R4 as best-fit substitution model selected by ModelFinder and nonparametric bootstrap branch support (100 replicates) [7, 16]. The average nucleotide identities (ANIs) of the genomes were calculated with FastANI (version 1.34) [17]. To demarcate taxon boundaries in the phylogenomic tree, we normalized the phylogenomic tree using the relative evolutionary divergence (RED) metric [18] and harmonized taxonomic ranks based on the specific RED values of neighboring taxa in the domain *Archaea* [7].

To analyze host distribution among intestinal lineages, the 16S rRNA gene sequences from relevant studies were imported into the DictDb 5.0 Archaea reference database [2]. Maximum-likelihood trees were calculated using IQ-TREE 2 with the substitution model GTR + I + G4 [16, 19]; tree topology was tested using maximum-parsimony analysis [20]. Node support was assessed using ultrafast bootstrap analysis (UFBoot2) [21].

### Comparative genomics

Only high-quality MAGs with at least 90% completeness and less than 5% contamination were used for comparative genome analysis. When lineages had only a small number of genomes, MAGs with slightly lower completeness (more than 85%) were included (see Table S1). Genome size, gene number, coding density, and GC content were determined using CheckM tools [22] or the NCBI prokaryotic genome annotation pipeline [23]. Pseudogenes were identified by the NCBI annotation pipeline and using Pseudofinder [24] on protein-coding genes predicted with Prokka v. 1.14.5 [25]. For the analysis of the metabolic pathways, annotation results were verified and missing functions were identified using Blast with a threshold *E*-value of 1e^−5^ and BlastKoala [26]. The annotation of amino acids biosynthesis pathways was refined with GapMind (Table S4); results for alanine, aspartate, or glutamate, which are easily formed by non-specific transaminations from common intermediates of central metabolism, were omitted [27].

Putative adhesin-like proteins and other proteins assumed to be involved in cell attachment were identified based on the automated annotation of NCBI. Specifically, we counted all coding regions annotated as Ig-like domain proteins [28], adhesins or adhesin-like domains [13, 14], eukaryote-like proteins tetratricopeptide, leucine-rich [29], right-handed beta-helical domains, beta strand repeat-containing proteins, and pseudomurein-binding repeats [30] (Table S3).

### Statistical analysis and data visualization

Data were statistically analyzed with R v3.5.1, and plots were generated with ggplot2 [31]. Differences between groups were tested with a Shapiro–Wilk normality test, followed by a Kruskal–Wallis *H*-test and a post hoc Dunn test with Holm *p*-value adjustment. The phylogenomic tree was visualized using iTOL v6.8 [32]. The 16S rRNA phylogenetic tree was visualized using ARB software package (v7.0) [33].

### Other aspects

The study did not involve euthanasia or anesthesia of animals.

## Results and discussion

### Phylogenomic analysis of *Methanobacteriota*

**Fig. 1:**
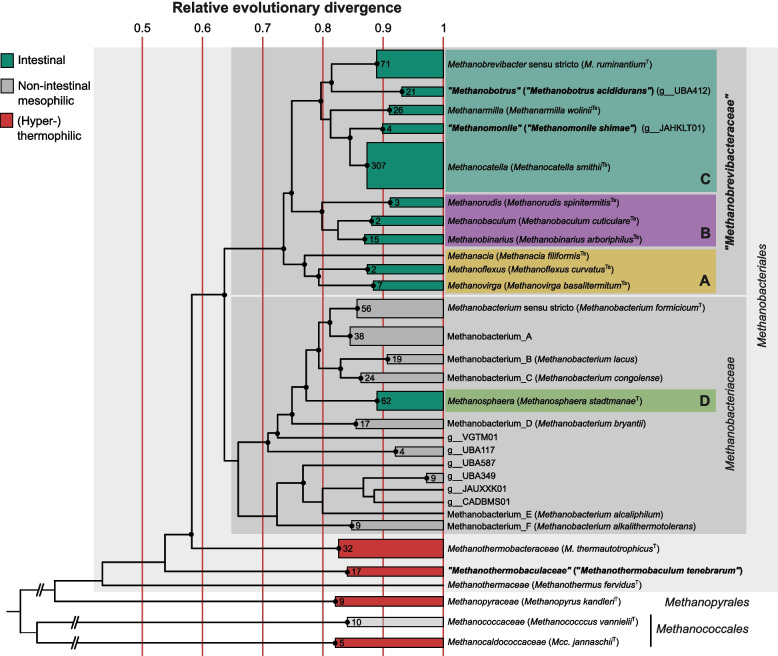
Phylogenomic tree of the phylum *Methanobacteriota*. Newly proposed taxa are shown in bold. Type species are given in parentheses; the index letters indicate ICNP (T) or SeqCode (Ts). The intestinal clades are highlighted. The maximum-likelihood tree was inferred from the concatenated alignment of 53 protein-coding genes and was normalized using relative evolutionary divergence (RED) values determined with PhyloRank. Bullets on the internal nodes indicate UFBoot support (● ≥ 95%, 1000 replicates). The values in the collapsed clades indicate the number of genomes included. An expanded version of the tree, including isolation sources, is shown in the Supplementary information (Fig. S1)

A comprehensive phylogenomic analysis of the entire phylum, including also high-quality MAGs of all genus-level lineages in GTDB, confirmed the monophyly of the classes *Methanobacteria*, *Methanococci*, and *Methanopyri*, each of which comprises only a single order (Fig. 1). The order *Methanobacteriales* consists of three family-level lineages, specifically *Methanothermaceae*, *Methanothermobacteraceae*, and *Methanobacteriaceae*. Since *Methanothermobacter tenebrarum* and its associated MAGs occupy a basal position (f__DSM-23052), which renders the family *Methanothermobacteraceae* polyphyletic, we propose to reclassify it as “*Methanothermobaculum tenebrarum”* in the new family “*Methanothermobaculaceae*”.

The family *Methanobacteriaceae* comprises all members of the genera *Methanobacterium*, *Methanosphaera*, and *Methanobrevibacter*. The genus *Methanobacterium* is polyphyletic with respect to *Methanosphaera*. The low RED values of the internal nodes reveal that *Methanobacterium* sensu lato consists of numerous genus-level lineages (Methanobacterium_A–F and several lineages without cultured representatives included in the framework of GTDB), indicating a future need for taxonomic revision. The same is true for the genus *Methanobrevibacter* sensu lato, which had been split into nine genus-level lineages already in a previous study [2]. A hitherto undescribed lineage (g__JAHKLT01) comprises the genome of “*Methanomonile shimae*” strain Ec1, which we recently isolated from the gut of the cockroach *Ergaula capucina*, two virtually identical single-cell amplified genomes (SCG-928-I08 and SCG-928-K11) from the cockroach *Periplaneta americana* [34], and a metagenome-amplified genome (MAG290) from an anaerobic digester (Table S1). A second hitherto undescribed lineage (g__UBA412) comprises the genome of *Methanobrevibacter acididurans* [35] obtained in the present study, which we propose to reclassify as “*Methanobotrus acididurans*”, and two MAGs from anaerobic digesters (UBA412, OH_PBB_124; Table S1). Based on the deep split between the highly supported *Methanobrevibacter* sensu lato clade and the remaining lineages of the family *Methanobacteriaceae* (RED value < 0.63; Fig. 1), we propose to reclassify all genera in this clade in the new family “*Methanobrevibacteraceae”*. The formal description of all new taxa under the rules of the ICNP will be addressed in a separate study.

The eleven genera in the family “*Methanobrevibacteraceae*” form three highly supported subfamily clades (Fig. 1). While clade A (*Methanacia*, *Methanoflexus*, and *Methanovirga*) consists exclusively of representatives from the termites, clade B (*Methanobaculum*, *Methanobinarius*, and *Methanorudis*) includes also representatives from the intestinal tract of other arthropods. Representatives of clade C occur either in the intestinal tract of vertebrates (*Methanobrevibacter* sensu stricto, *Methanarmilla*, and *Methanocatella*) or arthropods (*Methanomonile*), or in anaerobic digesters (*Methanobotrus*).

**Fig. 2:**
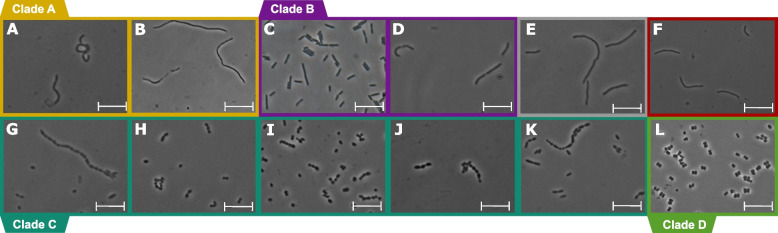
Phase-contrast photomicrographs of the type species of intestinal *Methanobacteriota*: *Methanoflexus curvatus* (**A**), *Methanacia filiformis* (**B**), *Methanobinarius arboriphilus* (**C**), *Methanobaculum cuticulare* (**D**), *Methanobrevibacter ruminantium* (**G**), “*Methanomonile shimae*” (**H**), “*Methanobotrus acididurans*” (**I**), *Methanarmilla wolinii* (**J**), *Methanocatella smithii* (**K**), and *Methanosphaera stadtmanae* (**L**). The non-intestinal mesophilic *Methanobacterium formicicum* (**E**) and thermophilic *Methanothermobacter marburgensis* (**F**) are shown for comparison. Agar-coated slide mounts were prepared from cultures in the early exponential phase [11]. All scale bars represent 5 μm

The subclades of “*Methanobrevibacteraceae*” differ significantly in cell morphology. Species in clades A and B have a rod-shaped or filamentous morphology, whereas representatives of clade C are cocci or coccoid rods (Fig. 2). Similar differences are found also in the family *Methanobacteriaceae*. Here, the coccoid members of the genus *Methanosphaera* (clade D) are embedded among members of *Methanobacterium* sensu lato (Fig. 1), which have a rod-shaped morphology (Fig. 2).

### Evolutionary history of the intestinal lineages

A specific association with the intestinal tract of animals occurred at least twice during the evolutionary radiation of the order *Methanobacteriales*, once in the genus *Methanosphaera* (*Methanobacteriaceae*), and once in a common ancestor of “*Methanobrevibacteraceae*”. Members of the genus *Methanobacterium*, which are found in various environments, are rare and inconsistently represented in the bovine rumen [3, 36]. Phylogeny and comparative genome analysis of the phylum *Methanobacteriota* indicate that the host-associated clades evolved from mesophilic, free-living, presumably autotrophic ancestors that conserved energy by CO_2_-reducing hydrogenotrophic methanogenesis. While members of the genus *Methanosphaera* occur exclusively in mammals [3], the family “*Methanobrevibacteraceae*” has a much broader host range and comprises lineages that are specific for either invertebrate or vertebrate host [2, 5, 6].

**Fig. 3:**
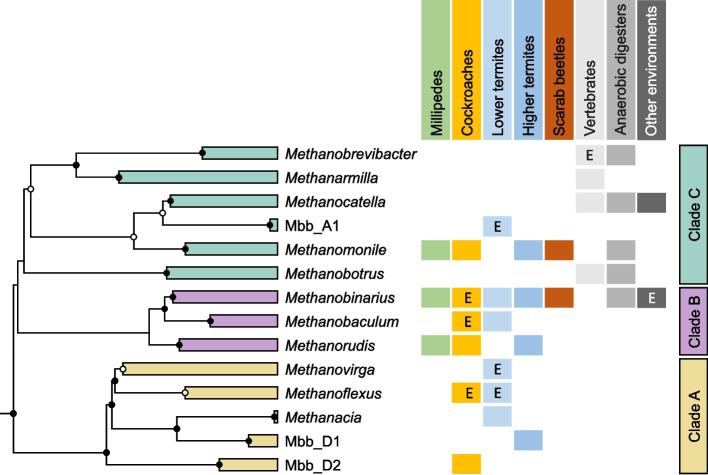
Distribution of genus-level lineages in the family “*Methanobrevibacteraceae*” among different host groups. The maximum-likelihood tree is based on a curated alignment of near-full-length 16S rRNA genes (> 1400 sites); bullets at the internal nodes indicate bootstrap support (filled ≥ 99%, open ≥ 95%; 1000 replicates each). The tree was re-rooted to reflect the topology of the genome tree (Fig. 1). The branches were justified; clades with putative endosymbionts (E) are labelled. A fully expanded version of the original tree is included in the Supplementary information (Fig. S11)

A detailed phylogenetic analysis of the 16S rRNA genes of “*Methanobrevibacteraceae*” recovered from different animals showed strong evidence for a coevolution with particular host groups (Fig. 3). Members of clades A and B are represented exclusively in arthropods. While members of clade B occur in a wide range of hosts, including termites, cockroaches, and millipedes, members of clade A are restricted to lower termites, with a specificity for particular termite families [2]. Members of the genus *Methanovirga* occur intracellularly in gut flagellates of lower termites [37–39] (Fig. S11), and genome analysis of the facultatively endosymbiotic representatives in *Cononympha* flagellates revealed specific adaptations to their hydrogen-rich intracellular environment [40]. Also certain members of the genera *Methanobinarius* and *Methanobaculum* are associated with termite gut flagellates [38], and members of the genus *Methanoflexus* have been shown to colonize anaerobic ciliates in the gut of cockroaches [41–43]. Members of the genera *Methanobaculum*, *Methanacia*, and *Methanoflexus* are also associated with the gut wall of termites [38, 44, 45] and possess adaptations that may facilitate colonization of the microoxic gut periphery (see below).

By contrast, most lineages in “*Methanobrevibacteraceae*” clade C are exclusively associated with vertebrates (Fig. 3). Previous high-throughput analyses of the gut microbiota of mammals already suggested that the genera common in ruminants (*Methanobrevibacter* sensu stricto, *Methanarmilla*, and *Methanocatella*) were independently acquired [3, 46]. While *Methanobrevibacter* sensu stricto seems to be restricted to ruminants [46], members of the genus *Methanarmilla* prevail also in rodents [3], and members of the genus *Methanocatella* are found also in other mammals and birds. Most species show a specificity for particular host groups, such as *Methanocatella smithii* and *Methanocatella intestini* for primates [47, 48]. Also the largely understudied gut microbiota of marsupials comprises members of the genera *Methanocatella* [49], including a new strain of *Methanocatella gottschalkii* recently isolated from a kangaroo [50].

The highly supported basal position of arthropod-associated lineages (clades A and B) in the radiation of “*Methanobrevibacteraceae*” (Fig. 1) is consistent with the assumption that the mammal-associated genera in clade C were derived from arthropod-associated ancestors. While the evolutionary transition of arthropods to a terrestrial lifestyle started during the Middle Cambrian (about 520 Ma) [51], mammals emerged only in the Middle Triassic (about 250 Ma) [52]. The intestinal methanogens of mammals were most likely acquired via predation or the accidental consumption of arthropods. This idea is supported by the observation that short-read sequences of *Methanobrevibacteraceae* from insectivorous mammals cluster with sequences from insects [3]. Representatives of the genera *Methanobotrus*, *Methanomonile*, and *Methanocatella* recovered from anaerobic digesters were most likely derived from gut-associated ancestors introduced with the inoculum or by fecal contaminations [53].

The entirely intestinal genus *Methanosphaera* (clade D) has evolved independently in the family *Methanobacteriaceae*. Its representatives were recovered exclusively from mammalian hosts, including ruminants, humans, and marsupials [49, 54, 55]. The origin of the genus among free-living lineages of the supergenus *Methanobacterium* is highly supported in phylogenomic analyses ([2], Fig. 1, Fig. S1), indicating that its sister position to “*Methanobrevibacteraceae*” in most 16S rRNA gene-based studies is artificial.

### Genomic and metabolic adaptations

**Fig. 4:**
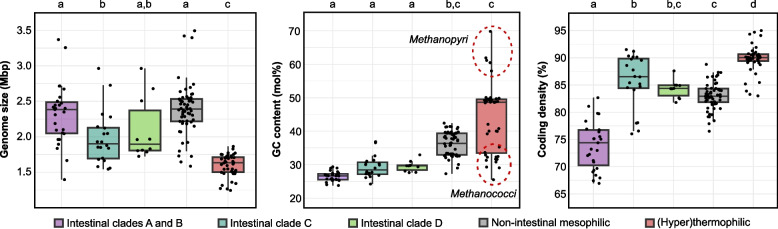
Genome characteristics of different ecological groups in the phylum *Methanobacteriota*. For group designations, see Figs. 1 and 2. Groups that share the same letter are not significantly different. For details, see Table S2

The smallest genomes in the phylum *Methanobacteriota* are found among the (hyper)thermophilic clades, which typically also have higher coding densities and lower numbers of pseudogenes than the other ecological groups. Such genome streamlining is common in extreme environments [56] and most likely caused by a strong purifying selection, as documented for marine oligotrophic bacteria [57]. Also intestinal *Methanobacteriota* generally show a reduced genome size, a lower coding density, and a higher percentage of pseudogenes than the mesophilic, free-living groups (Fig. 4, Table S2), which are symptoms of genome erosion [58]. A reduced genome size, particularly in non-ruminant hosts, has been documented already among members of the genus *Methanosphaera* [59]. However, the differences in genome size to the mesophilic non-intestinal group are significant only in case of the mammal-associated clade C. The highest number of pseudogenes is present in the arthropod-associated clades A and B, amounting to almost 14% of the overall gene content (Table S2). While the GC content of all intestinal groups is significantly lower than that of the non-intestinal mesophiles, the signal is taxonomically divergent in the (hyper)thermophilic lineages (on average, 46.6 mol% in *Methanobacteria*, 31.4 mol% in *Methanococci*, and 61.0 mol% in *Methanopyri*).

Genome erosion is promoted by a copiotrophic gut environment and the development of functional dependencies on other community members [60]. In *Methanobacteriota*, the associated gene losses resulted in auxotrophies for numerous growth factors (see below), which are likely favored by the relaxed selection pressure on genes whose products are already present in the environment, leading to anabolic erosion (“Black Queen hypothesis”; [61]). However, the genomes of the facultatively endosymbiotic *Methanovirga* spp. [40] and the putatively obligately endosymbiotic *Methanobinarius endosymbioticus* [41] are not significantly smaller than those of other “*Methanobrevibacteraceae*” (Table S2), indicating that their intracellular association occurred much more recently than that of the bacterial endosymbionts of termite gut flagellates [62]. Nevertheless, the coding density of their genomes is lower than that of other members of clades A and B (Fig. 4), and the number of pseudogenes is significantly increased ([40]; Table S2), indicating progressive genome erosion in the endosymbiotic lineages.

**Fig. 5:**
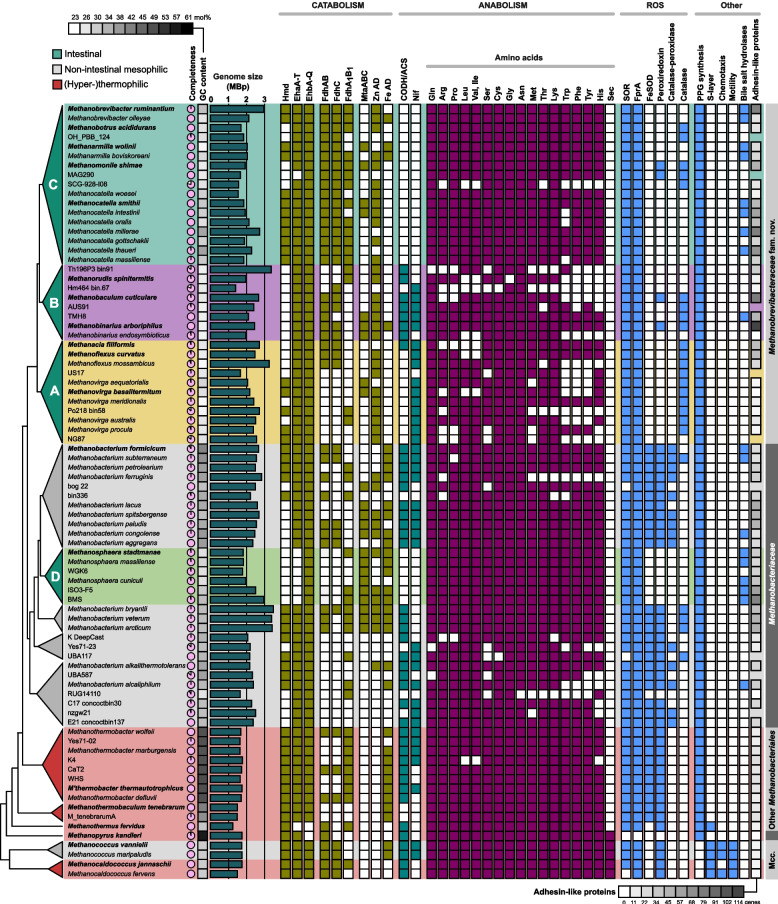
Metabolic characteristics of all species in the phylum *Methanobacteriota*, including uncultured lineages. Type species are marked in bold. The phylogenetic tree is schematic and not drawn to scale. The intestinal clades are highlighted. Genome size was estimated from assembly size and genome completeness. Abbreviations of enzyme names are explained in the text. Filled squares designate the presence in the genome of a particular gene or pathway. A fully expanded version of the tree is shown in Fig. S1; for a detailed annotation, including additional genomes, see Table S3

Despite the large variations in genome size and GC content among members of the phylum *Methanobacteriota*, numerous metabolic traits are highly conserved (Fig. 5). All genomes encode the full set of enzymes required to reduce CO_2_ to methane via the methyl branch of the Wood–Ljungdahl pathway, including a coenzyme F_420_-reducing [NiFe]-hydrogenase (FrhABG), a methyl-viologen-reducing [NiFe]-hydrogenase (MvhADG), an electron-bifurcating heterodisulfide reductase (HdrABC), and an energy-conserving methyl transferase complex (MtrA-H) (Table S3). The presence of other hydrogenase genes, however, differs between lineages. An Fe-only hydrogenase (Hmd), which plays a major role in methanogenesis under Ni-limiting conditions [63], is absent from many mesophilic clades, including all members of the genus *Methanosphaera*. The energy-converting [NiFe]-hydrogenase EhbA–Q is absent only from the genera *Methanothermus* and *Methanopyrus*, whereas the [NiFe]-hydrogenase EchA–F and the methanophenazine-reducing hydrogenase VhtACG, which are common in methanogens with cytochromes [64], are absent from all members of the phylum.

The presence of the Wood–Ljungdahl pathway is the basis for the ancestral traits of the *Methanobacteriota* phylum, i.e., hydrogenotrophic methanogenesis from H_2_ + CO_2_, chemiosmotic energy conservation via the MtrA-H complex, and autotrophic growth by the synthesis of acetyl-CoA from CO_2_ [65]. However, the transition to the copiotrophic gut environment affected the metabolism of all intestinal lineages. The most notable adaptation is found in the genus *Methanosphaera*, whose members have switched to an obligately methanol-dependent methanogenesis, conserving energy via a membrane-bound hydrogenase (EhbA–Q; [66]). Although the Wood–Ljungdahl pathway remained largely intact and provides intermediates for anabolism, all species have lost the ability to synthesize molybdopterin, the cofactor of formylmethanofuran dehydrogenases, and thus the ability for methanogenesis from CO_2_ ([67]; Table S3). Also, several other representatives of *Methanobacteriaceae* and “*Methanobrevibacteraceae*” encode a methanol methyltransferase complex MtaABC (Fig. 5). However, the capacity to grow on H_2_ + methanol has been demonstrated so far only for *Methanobacterium lacus* and *Methanobacterium veterum* [68, 69] (Table S1). The presence of MtaABC in “*Methanobrevibacteraceae*” was first noticed in *M. smithii* [70] and later also in other taxa [6, 71], but among the species that encode homologs of the complex (Fig. 5; Table S3), only *Methanarmilla wolinii* has been tested for growth on H_2_ + methanol, albeit with negative results [72].

The switch to a methyl-reducing methanogenesis in *Methanosphaera* was facilitated by the ancestral presence of a methanol methyltransferase complex (MtaABC) in *Methanobacteriales*, which is absent from all other lineages of *Methanobacteriota* (Fig. 5). Phylogenetic analysis revealed that it forms a separate branch that is most closely related to homologs from methanol-utilizing methanogens in other phyla (Fig. S2). It has been hypothesized that the capacity to grow on H_2_ + methanol provides a competitive advantage in intestinal environments that provide both substrates [3]. Methyl-reducing hydrogenotrophic methanogenesis becomes energetically advantageous over CO_2_-reducing methanogenesis at lower H_2_ partial pressures [73], which is of particular importance in microhabitats where H_2_ is limiting, such as the hindgut wall of termites and cockroaches [74, 75].

The presence of an F_420_-dependent formate dehydrogenase (FdhAB) and the corresponding formate transporter (FdhC) allows hydrogenotrophic methanogens to utilize formate as an alternative electron donor. However, the distribution of FdhAB varies between lineages (Fig. 5). Homologs are present in all members of “*Methanobrevibacteraceae*” except *Methanorudis* and *Methanovirga*, in several species of *Methanobacteriaceae* (but not in *Methanosphaera*), and in a few thermophiles. Only *Methanopyri* and *Methanococci* have the selenocysteine-containing variant of FdhA, which is consistent with the absence of the selenocysteine pathway in *Methanobacteria* (Table S3). FdhA is commonly present in two forms: a standard form with 680–700 amino acid (AA) residues, and a truncated homolog (FdhA_1_) with 360–380 AA residues, which is involved in methanogenesis under Ni-limiting conditions [76]; both forms are found throughout *Methanobacteriota* but not in all lineages (Table S3). In addition, several *Methanobacterium* and *Methanothermobacter* species possess an NADP-dependent formate dehydrogenase of bacterial origin (Table S3). The digestive tracts of both arthropods and ruminants contain appreciable amounts of formate [75, 77]. Although the majority of “*Methanobrevibacteraceae*” encodes FdhABC and should have the capacity to use formate as an additional electron donor, growth on formate has been documented so far only for two species (*Methanobaculum cuticulare* and *Methanobinarius arboriphilus* [44, 78]). The pseudogenization and subsequent elimination of *fdhABC* in members of the genus *Methanovirga* may be an adaptation to the absence of formate in their intracellular environment [40].

Many species in the *Methanobacteriota* phylum encode at least one type of NADP-dependent alcohol dehydrogenase (Fig. 5, Table S3). Thermophilic lineages have only an iron-containing variant, while the mesophilic lineages also possess a Zn-containing variant; both are of bacterial origin (Fig. S3, S4). The iron-containing variants present in the intestinal lineages were independently acquired from different bacterial sources. While *M. palustre* and *M. movilense* produce methane with 2-propanol and 2-butanol as electron donor [79, 80], *M. congolense* and *M. ferruginis* utilize also cyclopentanol [81, 82]. By contrast, strains of *Methanosphaera* and *Methanarmilla boviskoreani* [49, 83] oxidize ethanol with an NADP-dependent alcohol dehydrogenase. Our dataset documents a previously unknown diversity of NADP-dependent alcohol dehydrogenases in *Methanobacteriota*, which includes iron-containing and zinc-containing enzymes (Fig. 5, Table S3). Since members of the ethanol-utilizing genera *Methanarmilla* and *Methanosphaera* possess only the iron-containing enzyme (Fig. S3), it is possible that the Zn-containing enzyme, which was originally detected in *Methanobacterium* [84], is specific for secondary alcohols.

### Convergent losses of biosynthetic potential

While the pathway for the biosynthesis of cofactor F_430_, which is essential for methanogenesis, is present in all members of *Methanobacteriota*, they generally lack the capacity to synthesize heme from uroporphyrinogen III, the common precursor of all tetrapyrroles. With the exception of *Methanobrevibacter ruminantium* PS^T^ and one MAG from the genus *Methanorudis* (Th196P4 bin56) (Table S3), all species synthesize coenzyme M via the classical pathway [85]. By contrast, a CODH/ACS complex, which allows the synthesis of acetyl-CoA from CO_2_ and enables most free-living members of *Methanobacteriota* to grow autotrophically on H_2_ + CO_2_, is absent from almost all intestinal species ([6]; Fig. 5). Notable exceptions are members of clade B and *Methanacia filiformis*, which is consistent with the lack of acetate requirement in the respective isolates (Tables S1 and S3).

All members of *Methanobacteriota* possess an ammonium transporter, and most free-living lineages encode the complete pathways for the de novo biosynthesis of all proteinogenic amino acids (Fig. 5; Tables S3 and S4). However, the capacity to synthesize amino acids varies among the intestinal lineages. While almost all pathways are complete in the vertebrate-associated clades C and D, most species in the arthropod-associated clades A and B lack the capacity to synthesize certain amino acids. Moreover, most intestinal isolates require growth factors such as yeast extract, rumen fluid, and vitamins (Table 1, Table S1), suggesting that the loss of anabolic pathways is common in all intestinal clades*.* It has been hypothesized that the dependence on dietary components provided by the host and/or other gut microbiota promotes a vertical transfer of intestinal symbionts and limits their dispersal in environments that do not provide these essential nutrients [86].

The presence of *nifHDKE(N/B*), the minimal gene set required for the expression of a functional nitrogenase complex in *Methanococci* [87, 88] among many other members of the phylum indicates that the capacity for nitrogen fixation is widely distributed among both free-living and intestinal lineages of *Methanobacteriota* (Fig. 5). However, a *nif* operon is absent from all members of “*Methanobrevibacteraceae*” clade C and the genus *Methanosphaera*. Based on NifH phylogeny, the *nif* genes of *Methanobacteria* and *Methanococci* form monophyletic clades among the molybdenum-dependent nitrogenases of group III and are well separated both from their bacterial homologs and from those of other nitrogen-fixing methanogens (*Methanosarcinales* and *Methanomicrobiales*) (Fig. S5), which is in agreement with previous results [89]. The conservation of nitrogen fixation in the arthropod-associated clades A and B is consistent with the presence of diazotrophs also among the bacterial gut microbiota of termites and other lignocellulose-digesting arthropods with a nitrogen-poor diet [90]. Conversely, the convergent loss of nitrogenase in the vertebrate-associated clades C and D is most likely a response to the nutrient-rich environment provided by the gut of vertebrates, strengthening the hypothesis that environmental factors drive genome evolution in the intestinal lineages of archaea. This agrees with the absence of nitrogenases in the genus *Methanomonile*, the only member of clade B that is associated with omnivorous cockroaches, whose diet is more nitrogen-rich than that of wood-feeding termites.

### Adaptations to molecular oxygen and other traits

All life forms, including strict anaerobes, have evolved mechanisms protecting against molecular oxygen and reactive oxygen species (ROS) [91]. With only a few exceptions, members of *Methanobacteriota* encode homologs of superoxide reductase (SOR), F_420_H_2_ oxidase (FprA), rubrerythrin (FprB), and rubredoxin (FprC) ([92, 93]; Fig. 5; Table S3). Among non-intestinal lineages, the FprABC gene cluster is often associated with genes encoding an iron superoxide dismutase (FeSOD) and peroxiredoxin. FeSOD is of the archaeal type (Fig. S6) and was detected only in members of *Methanobacteriales*, whereas peroxiredoxin is also present in *Methanococci*. A Cu-ZnSOD or a NiSOD was not detected. A catalase was detected in many representatives of “*Methanobrevibacteraceae*”, whereas free-living *Methanobacteriaceae* (but not the intestinal *Methanosphaera*) possess either a catalase or a bifunctional catalase-peroxidase (Fig. 5, Table S3). Phylogenetic analysis revealed that both enzymes are of bacterial origin, but the donors differ from those of the catalases acquired by methanogens from the phylum *Halobacteriota* (Figs. S7 and S8).

An iron-dependent superoxide dismutase (FeSOD) was previously detected in only two species of *Methanobacteriota*, specifically *Methanobacterium bryantii* and *Methanothermobacter thermoautotrophicus* [94, 95]. Our data shows that FeSOD is present only in free-living *Methanobacteriales* but absent from all intestinal lineages (Fig. 5; Table S3). Since FeSODs of *Methanobacteriales* and other archaea are distinct from their bacterial homologs, the enzyme was probably present already in the common archaeal ancestor and subsequently lost in *Methanopyrales*, *Methanococcales*, and intestinal *Methanobacteriales* (Fig. S6).

Although the genes encoding FprA, rubrerythrin, rubredoxin, FeSOD, and peroxiredoxin are often found in the same gene cluster, indicating an orchestrated response against O_2_ and ROS (Table S3), FeSOD, peroxiredoxin and catalase-peroxidase are conspicuously absent from all intestinal lineages (“*Methanobrevibacteraceae*” and *Methanosphaera*; Fig. 5). However, a monofunctional catalase of bacterial origin, which is present in many members of extant “*Methanobrevibacteraceae*” (Figs. 5, and S7), was mostly likely acquired before the split of intestinal and non-intestinal lineages. Since members of *Methanobacteriota* cannot synthesize heme (this study), the production of an active enzyme by *Methanobinarius arboriphilus* requires the addition of hemin to the growth medium [96], and exogenous hemin has a positive effect on the resistance of *Methanobinarius arboriphilus* to oxidative stress [97]. Since hemin is relatively stable in anoxic environments, “*Methanobrevibacteraceae*” probably depend on other gut community members to provide heme, as postulated also for arthropod-associated spirochetes [98, 99].

The concomitant loss of FeSOD, peroxiredoxin, and catalase-peroxidase in “*Methanobrevibacteraceae*” and *Methanosphaera* also suggests that the gut environment shields intestinal methanogens from O_2_ and ROS. However, the presence of catalase differentiates arthropod-associated from vertebrate-associated species. Arthropod guts are much smaller than vertebrate guts, and the higher surface-to-volume ratio increases epithelial oxygen fluxes by several orders of magnitude [90]. Despite the constant influx of oxygen, the hindgut wall of many termites and cockroaches is colonized by members of “*Methanobrevibacteraceae*”. After the discovery of FprA in *Methanobinarius arboriphilus* and its role in the hydrogen-dependent reduction of O_2_ to water [92], it has been suggested that FprA is responsible for the high rates of O_2_ reduction by cell suspensions of *Methanobinarius arboriphilus* and *Methanobaculum cuticulare* [100]. The presence of FprA and the retention of catalase in all arthropod-associated species of “*Methanobrevibacteraceae*” most likely represent an adaptation to the exposure to O_2_ and the associated production of H_2_O_2_ by SOR. Notably, also *Methanimicrococcus* spp. and other *Methanosarcinaceae* colonizing the gut wall of arthropods exhibit catalase activity and possess a similar set of ROS protection enzymes [11].

In the context of the colonization of intestinal surfaces, it is important to consider that representatives of intestinal *Methanobacteriota* have been shown to possess various adhesin-like proteins (ALPs), which are considered to play a role in the physical interactions of intestinal *Methanobacteriota* with their mammalian, bacterial, or protistan partners [13, 67, 101]. In accordance with previous findings [14], many genomes of the intestinal lineages encode numerous ALPs and other proteins putatively involved in cell attachment (Fig. 5, Tables S3 and S3a). Notably, the number of ALPs encoded by the endosymbiotic genus *Methanovirga* was much lower than that of other “*Methanobrevibacteraceae*”, which may be an indication of adaptation to the endosymbiotic lifestyle. Moreover, ALP genes were abundantly represented also in many non-intestinal *Methanobacteriaceae* and, in much lower abundance, in other *Methanobacteriota*. Our analysis not only confirmed the large proportion of ALP genes among “*Methanobrevibacteraceae*” and *Methanosphaera* observed in previous studies [14, 30], but also demonstrated that genes encoding ALPs and other proteins assumed to be involved in cell attachment are distributed across the entire phylum, albeit their number varies between lineages (Fig. 5). Notably, the predominant ALPs in the intestinal lineages were not common outside *Methanobacteriaceae* and “*Methanobrevibacteraceae*”, whereas sequences annotated as tetratricopeptides and pseudomurein-binding domains occurred in most members of the phylum (Table S3a).

The presence of bile salt hydrolases (BSH) is considered an adaptive trait among the bacterial gut microbiota of vertebrates [102]. Consistent with this assumption, homologs of BSH genes are found mostly in the mammal-associated lineages of clades C and D, but not in all species, suggesting that these methanogens are protected from bile salts by other community members. Phylogenetic analysis revealed that the homologs from “*Methanobrevibacteraceae*” and *Methanosphaera* cluster among representatives of *Lactobacillales*, whereas the few homologs from *Methanobacterium* spp. cluster among *Clostridiales* (Fig. S9), confirming that the BSH genes of archaea were acquired by horizontal gene transfer from bacterial donors [14].

Consistent with the presence of a cell envelope with a proteinaceous surface layer in *Methanococci* [103], we identified S-layer proteins in all genomes of this class (Fig. 5). They are most closely related to the S-layer proteins of *Thermococcales*, the closest relatives of *Methanobacteriota* (Methanobacteriota_B in the GTDB classification) (Fig. S10), corroborating the common ancestry of this trait. With the exception of *Methanothermus fervidus* [104], all other members of *Methanobacteriota* lack an S-layer. Instead, they synthesize pseudopeptidoglycan (PPG), also known as pseudomurein, a unique cell-wall component of *Methanobacteria* and *Methanopyri* (Fig. 5), whose biosynthetic pathway was most likely acquired from the bacterial domain [105, 106]. Although the pathway remains to be fully elucidated, all genes presently known to be involved in PPG biosynthesis are present (Table S3). Notably, members of the genus *Methanothermus* (class *Methanobacteria*) possess both pseudopeptidoglycan and S-layer proteins [107], but the S-layer protein is not homologous to that of *Methanococci*.

A gene cluster encoding the synthesis of archaella, which are responsible for swimming motility in many archaea [108], was detected exclusively in representatives of the class *Methanococci* (Fig. 5; Table S3). This is consistent with the general lack of motility in *Methanobacteria* [109] and suggests an early loss of archaella in the evolution of *Methanobacteria* and *Methanopyri*. Notably, both *Methanopyrus kandleri* and *Methanothermus fervidus* reportedly possess tufts of polar flagella [110, 111]. Although *M. kandleri* has been described as motile, the only genes of the archaellum cluster present in both organisms are FlaI and FlaJ (Table S3). The exclusive presence of a chemotaxis system CheBCDFRWY [112] in members of *Methanococci* (Table S3) is in agreement with the lack of motility in other *Methanobacteriota*.

## Conclusion

*Methanobacteriota* provide an excellent example of how the transition from a free-living lifestyle to an intestinal environment drives genome evolution in host-associated archaea. As in their bacterial counterparts, the transition was accompanied by genome erosion and a sharp decrease in coding density, leading to a loss of biosynthetic capacities. While most of the changes in anabolism are clearly convergent in different lineages, others—such as the conservation of the pathways for acetyl-CoA biosynthesis and nitrogen fixation—are likely affected by host diet and ecosystem services of other microbiota. It is remarkable that the transition to a host-associated environment in *Methanobacteriaceae* has caused the same shift from a CO_2_-reducing to an exclusively methyl-reducing methanogenesis as previously found in *Methanosarcinaceae* (*Halobacteriota*) [11, 12], which demonstrates that environmental factors serve as evolutionary drivers across the archaeal domain, and that both catabolic and anabolic processes are aligned with the environment to increase fitness.

## Supplementary Information

Additional file 1. Figure S1. Phylogenomic tree of selected *Methanobacteriota* (expanded version of the tree in Fig. 1, showing all species and the isolation sources of the respective type strains). Figure S2. Phylogenetic tree of methanol methyltransferase (MtaB) proteins from various prokaryotes. Figure S3. Phylogenetic tree of iron-dependent alcohol dehydrogenase from *Methanobacteriota*and other prokaryotes. Figure S4. Phylogenetic tree of the zinc-dependent alcohol dehydrogenase of *Methanobacteriota*and other prokaryotes. Figure S5. Phylogenetic tree of NifH and related proteins (Nif groups IV to VI). Figure S6. Phylogenetic tree of superoxide dismutase of *Methanobacteriota*. Figure S7. Phylogenetic tree of the heme-containing monofunctional catalase of *Methanobacteriota*. Figure S8. Phylogenetic tree of the heme-containing bifunctional catalase-peroxidase of *Methanobacteriota*. Figure S9. Phylogenetic tree of the bile hydrolases of *Methanobacteriota*. Figure S10. Phylogenetic tree of the S-layer proteins of *Methanobacteriota*. Figure S11. Phylogenetic 16S rRNA gene tree illustrating the relationship between various lineages of “*Methanobrevibacteraceae*” (expanded version of the tree in Fig. 4, showing accession numbers and isolation sources for sequences).

Additional file 2. Table S1. Characteristics of the *Methanobacteriota* genomes included in this study. Table S2. Properties of selected *Methanobacteriota* genomes and their statistical analysis. (data visualized in Fig. 4).

Additional file 3. Table S3. Annotations of genes encoding metabolic pathways and/or functional markers of metabolic properties for selected genomes of *Methanobacteriota*. Details on the distribution of adhesin-like proteins and other proteins assumed to be involved in cell attachment are shown in Table 3a.

Additional file 4. Table S4. Annotations of genes encoding amino acid biosynthetic pathways in selected *Methanobacteriota* genomes.

## Data Availability

The accession numbers of the genome sequences of obtained in this study are freely available at NCBI (*M. acididurans*, GCF_046197875; *M. shimae*, GCF_046197865). All previously published genomes are available at NCBI (accession numbers are given in Table S1). The MAGs taken from the study Arora, Kinjo, Šobotník, et al., *Microbiome* 10, 78 (2022) can be downloaded from Figshare (10.6084/m9.figshare.17031746). No special codes or scripts were used in this study.
