# Supplementary material for: Convergent evolution of intestinal lineages in the phylum Methanobacteriota
Source: Microbiome. 2026 May 2;14:130. doi: 10.1186/s40168-026-02434-5 (PMC13135261; doi:10.1186/s40168-026-02434-5)
Supplement: Supplementary file 1 — Additional file 1. Figure S1. Phylogenomic tree of selected Methanobacteriota (expanded version of the tree in Fig. 1, showing all species and the isolation sources of the respective type strains). Figure S2. Phylogenetic tree of methanol methyltransferase (MtaB) proteins from various prokaryotes. Figure S3. Phylogenetic tree of iron-dependent alcohol dehydrogenase from Methanobacteriotaand other prokaryotes. Figure S4. Phylogenetic tree of the zinc-dependent alcohol dehydrogenase of Methanobacteriotaand other prokaryotes. Figure S5. Phylogenetic tree of NifH and related proteins (Nif groups IV to VI). Figure S6. Phylogenetic tree of superoxide dismutase of Methanobacteriota. Figure S7. Phylogenetic tree of the heme-containing monofunctional catalase of Methanobacteriota. Figure S8. Phylogenetic tree of the heme-containing bifunctional catalase-peroxidase of Methanobacteriota. Figure S9. Phylogenetic tree of the bile hydrolases of Methanobacteriota. Figure S10. Phylogenetic tree of the S-layer proteins of Methanobacteriota. Figure S11. Phylogenetic 16S rRNA gene tree illustrating the relationship between various lineages of “Methanobrevibacteraceae” (expanded version of the tree in Fig. 4, showing accession numbers and isolation sources for sequences). [file 40168_2026_2434_MOESM1_ESM.pdf]

Supplementary figures

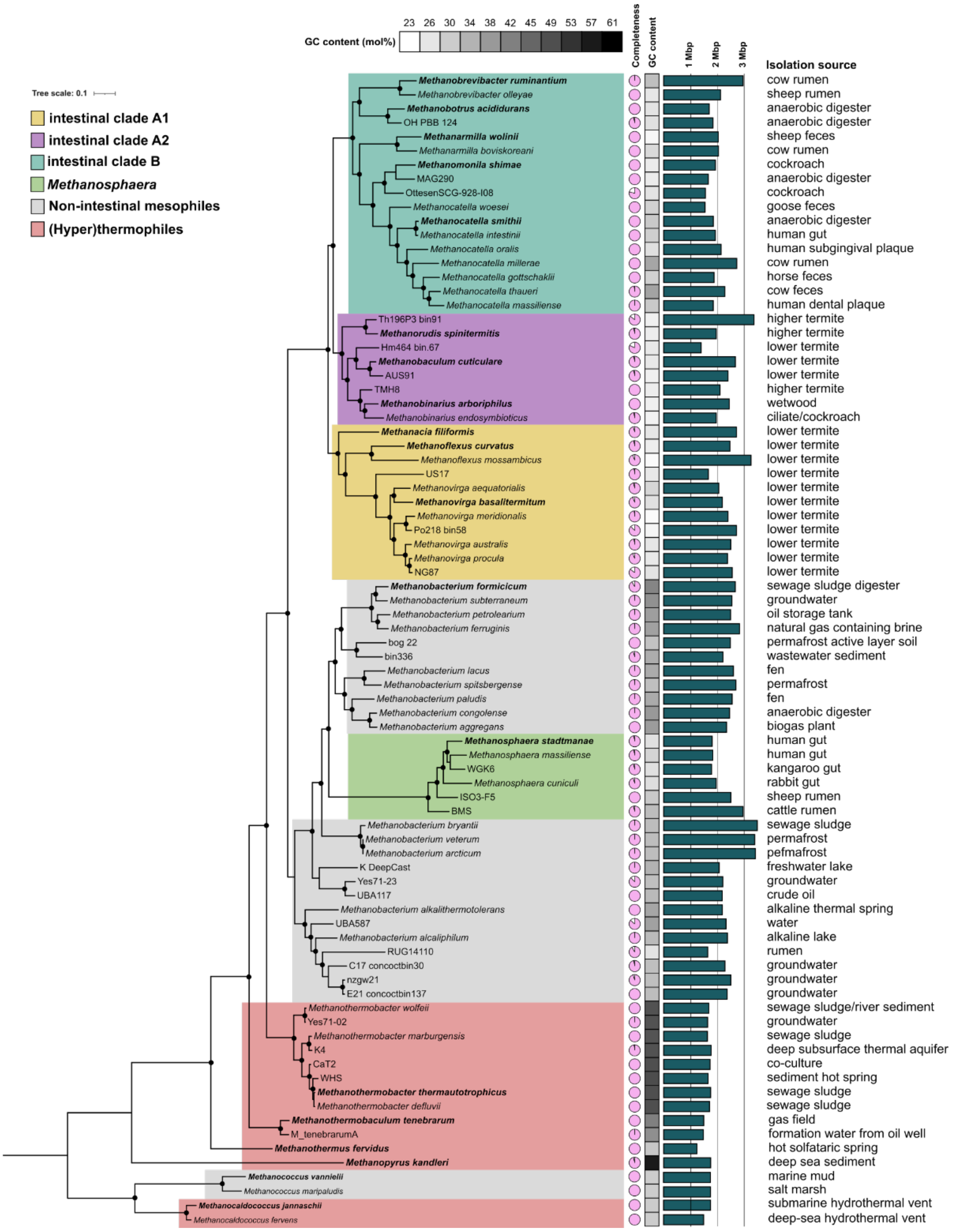

Figure S1. Phylogenomic tree of selected *Methanobacteriota*. This is an expanded version of the tree in Figure 2, showing all species and the isolation sources of the respective type strains. The scale bar indicates the number of substitutions per site. The tree was rooted using *Thermococcales* as outgroup. Type species are marked in bold. For genome accession numbers, see Table S1.

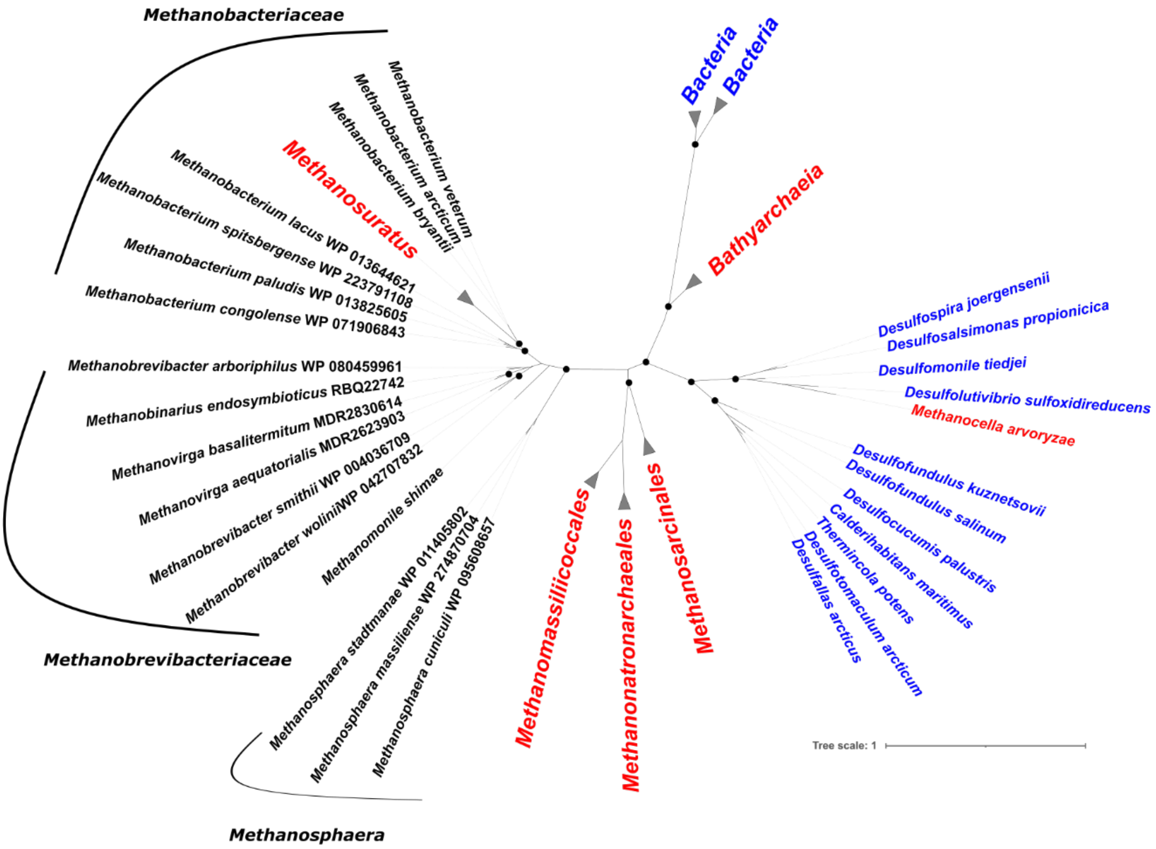

Figure S2. Phylogenetic tree of methanol methyltransferase (MtaB) proteins from various prokaryotes. Colors indicate homologs from intestinal *Methanobacteriota* (black), other archaea (red), and bacteria (blue). The maximum-likelihood tree was generated using IQ-TREE under the LG+I+G4 model of evolution. Bullets on the internal nodes indicate UFBoot support (● ≥ 95%, 1000 replicates). The scale bar indicates the number of substitutions per site.

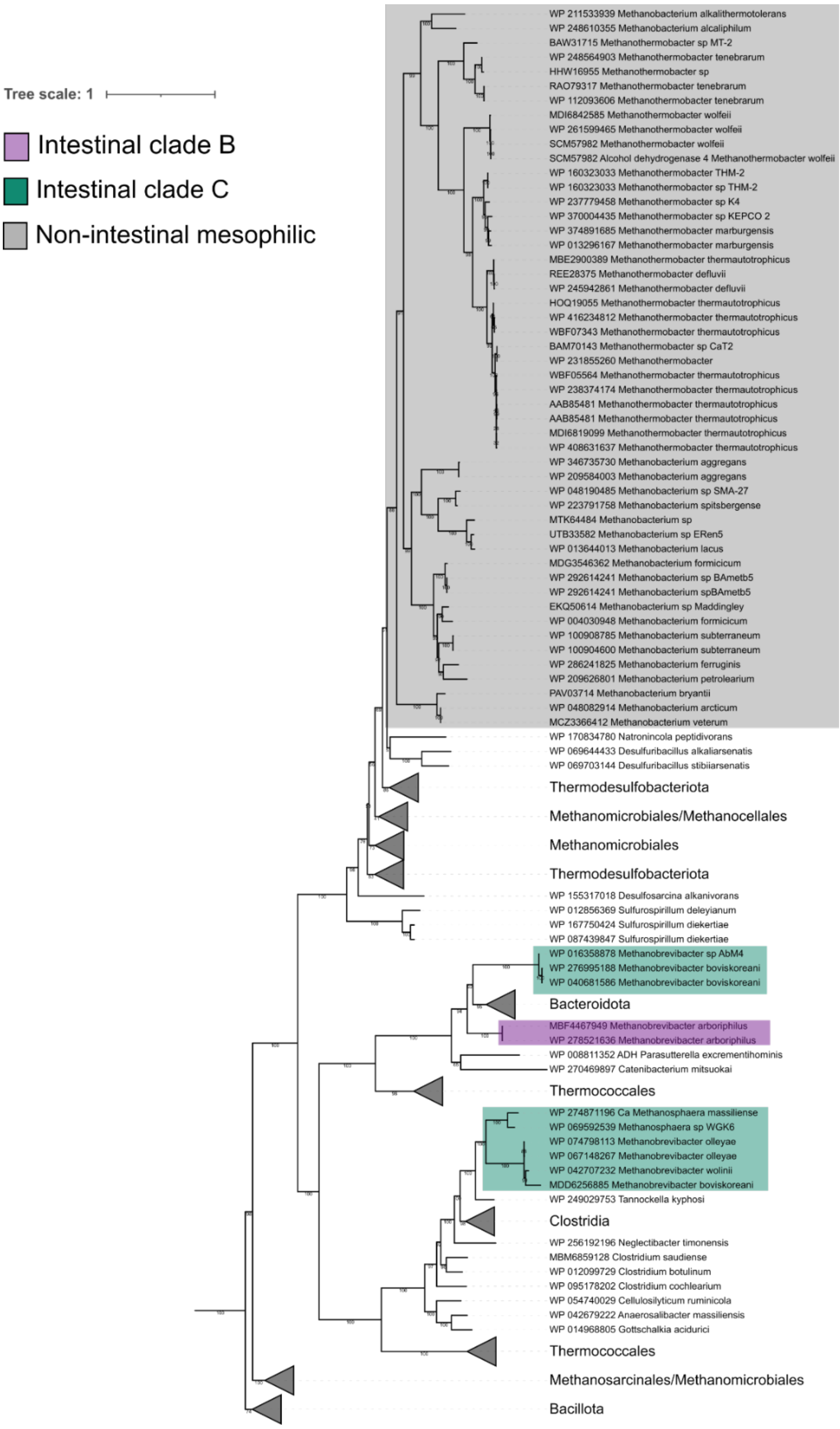

Figure S3. Phylogenetic tree of iron-dependent alcohol dehydrogenase from *Methanobacteriota* and other prokaryotes. The maximum-likelihood tree was generated using IQ-TREE under the LG+I+G4 model of evolution. Values at the internal nodes indicate UFBoot support (1000 replicates). The scale bar indicates the number of substitutions per site.

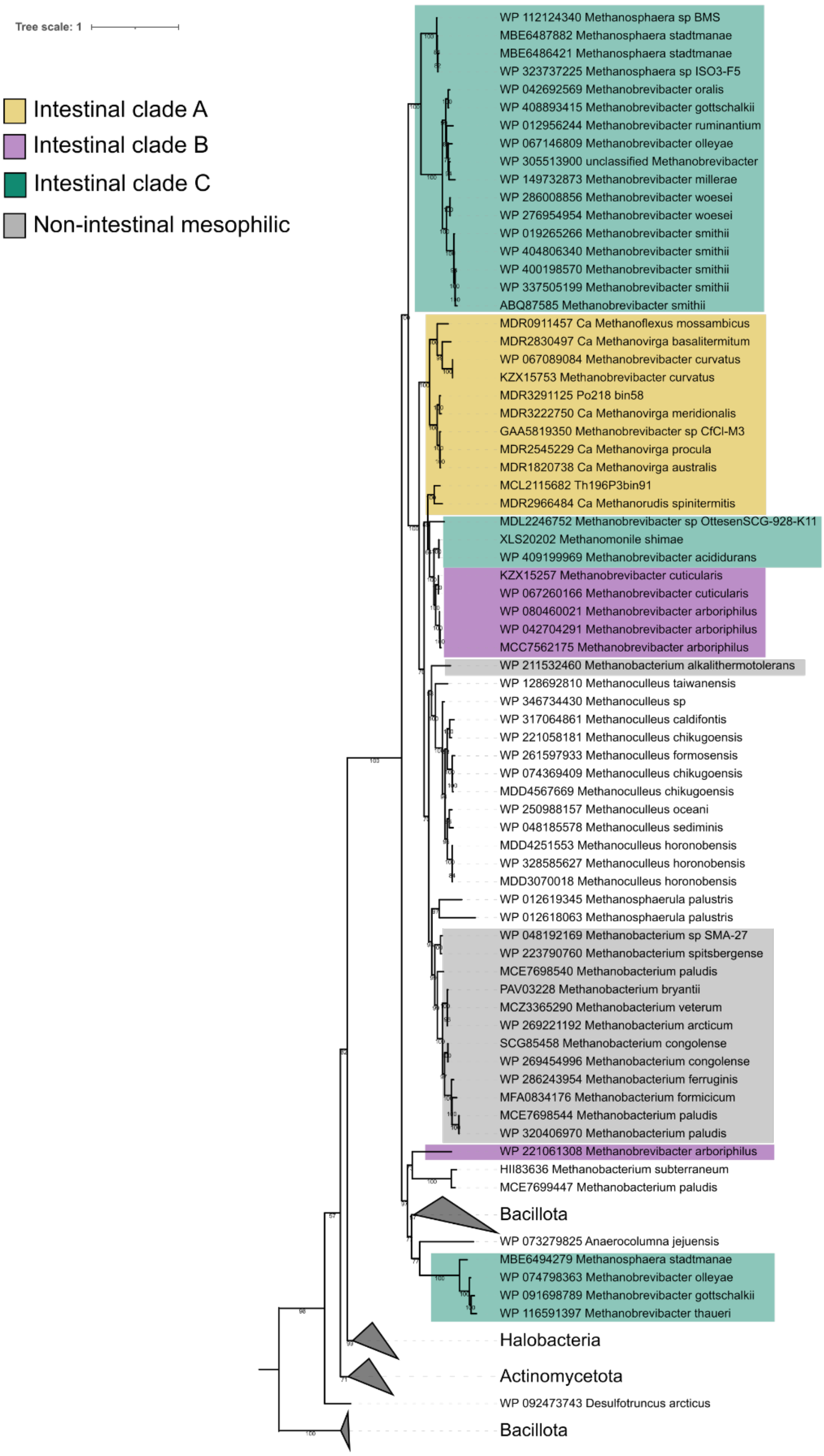

Figure S4. Phylogenetic tree of the zinc-dependent alcohol dehydrogenase of *Methanobacteriota* and other prokaryotes. The maximum-likelihood tree was generated using IQ-TREE under the LG+I+G4 model of evolution. Values at the internal nodes indicate UFBoot support (1000 replicates). The scale bar indicates the number of substitutions per site.

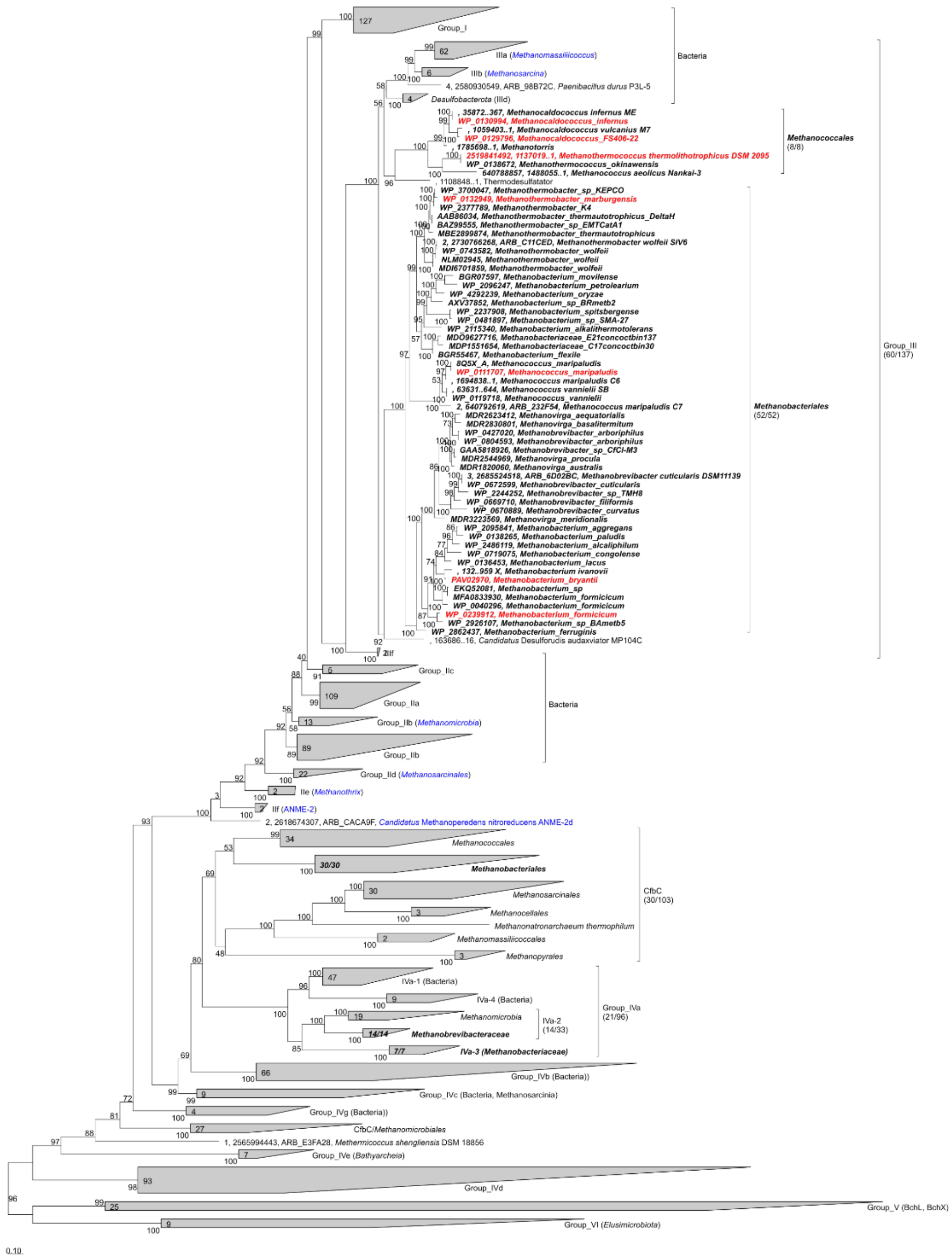

Figure S5. Phylogenetic tree of NifH and related proteins (Nif groups IV to VI). Homologs from *Methanobacteriota* are marked in bold; species with the experimentally confirmed ability to fix N_2 are highlighted in red. NifH homologs from other methanogens are marked in blue. Homologs of CfbC and other proteins (groups IV to VI) were included as outgroup. The maximum-likelihood tree is based on a manually curated AA alignment and was generated using IQ-TREE under the LG+I+G4 model of evolution. Values at the nodes indicate UFBoot support (1000 replicates). The scale bar indicates the number of substitutions per site.

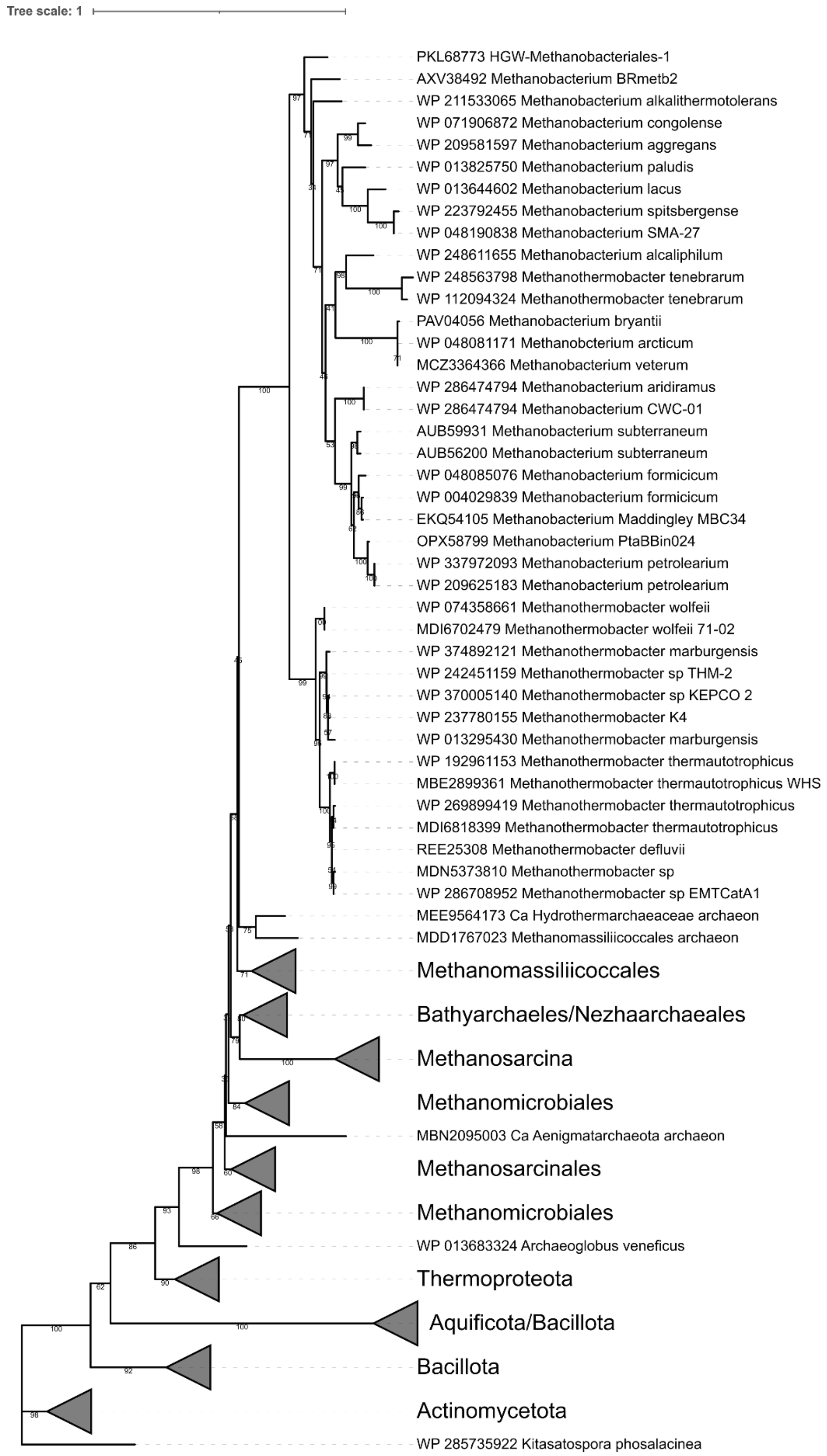

Figure S6. Phylogenetic tree of superoxide dismutase of *Methanobacteriota*. The maximum-likelihood tree was generated using IQ-TREE under the LG+I+G4 model of evolution. Values at the nodes indicate UFBoot support (1000 replicates). The scale bar indicates the number of substitutions per site.

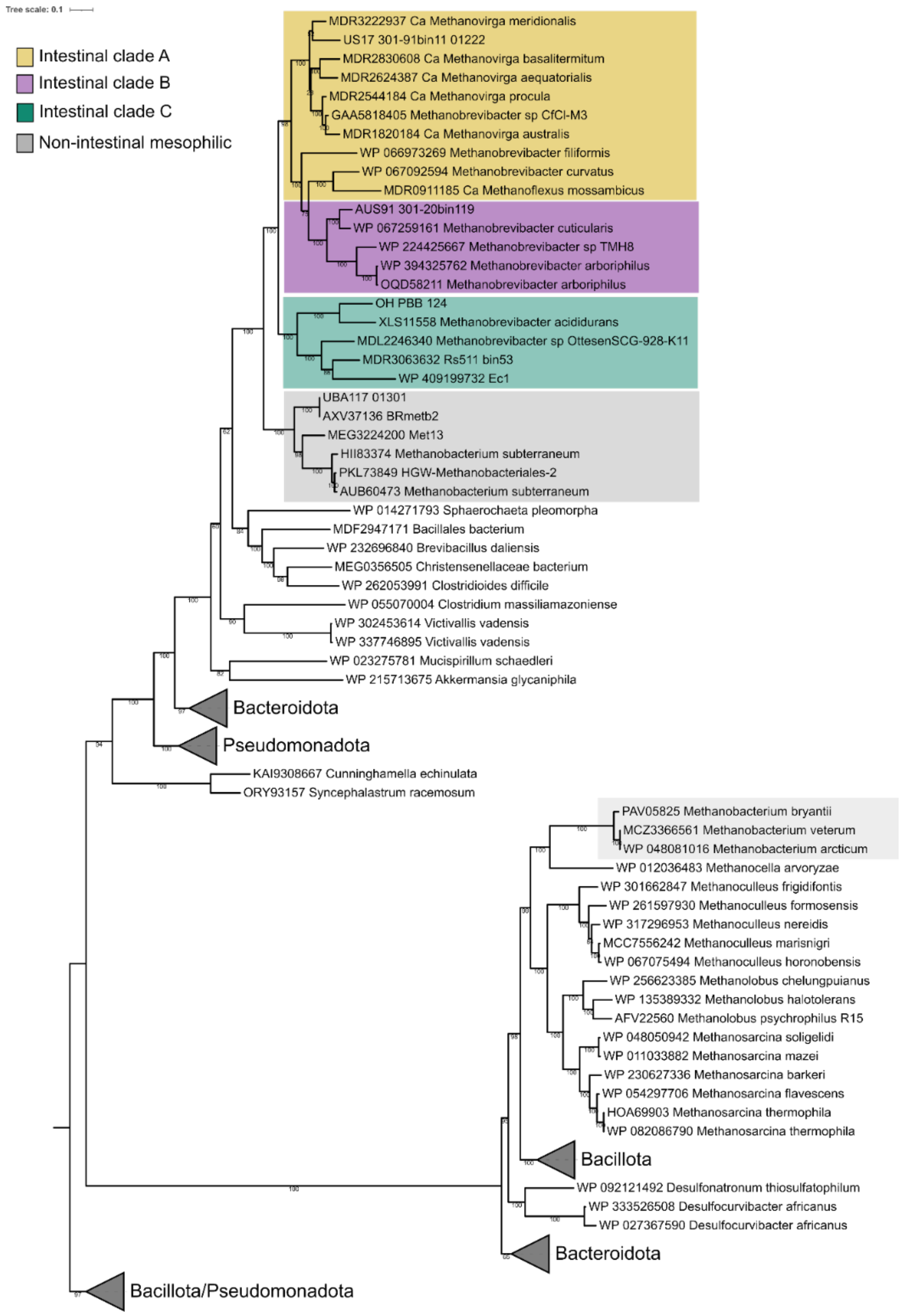

Figure S7. Phylogenetic tree of the heme-containing monofunctional catalase of *Methanobacteriota*. The maximum-likelihood tree was generated using IQ-TREE under the LG+I+G4 model of evolution. Values at the nodes indicate UFBoot support (1000 replicates). The scale bar indicates the number of substitutions per site.

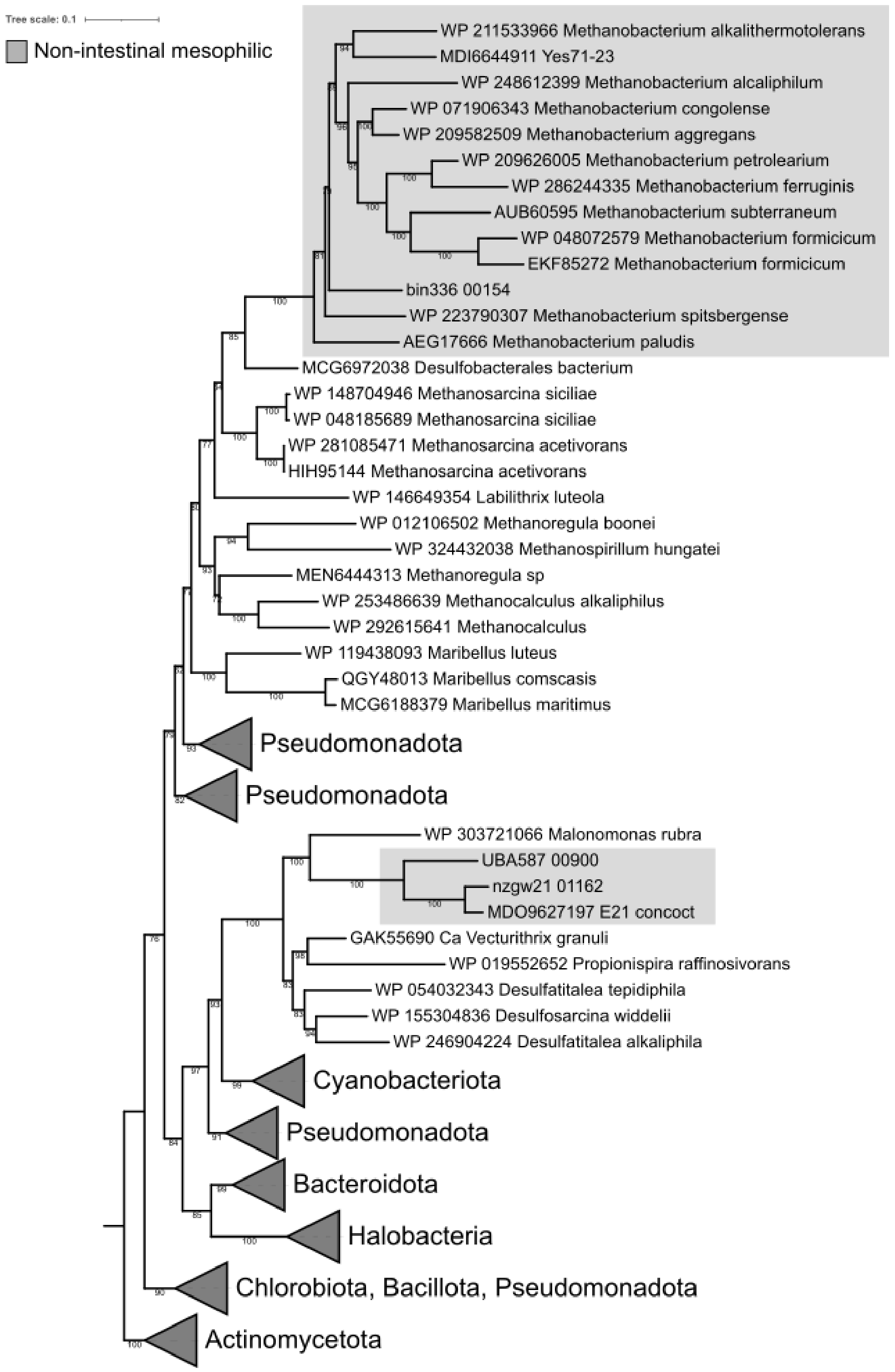

Figure S8. Phylogenetic tree of the heme-containing bifunctional catalase-peroxidase of *Methanobacteriota*. The maximum-likelihood tree was generated using IQ-TREE under the LG+I+G4 model of evolution. Values at the nodes indicate UFBoot support (1000 replicates). The scale bar indicates the number of the substitutions per site.

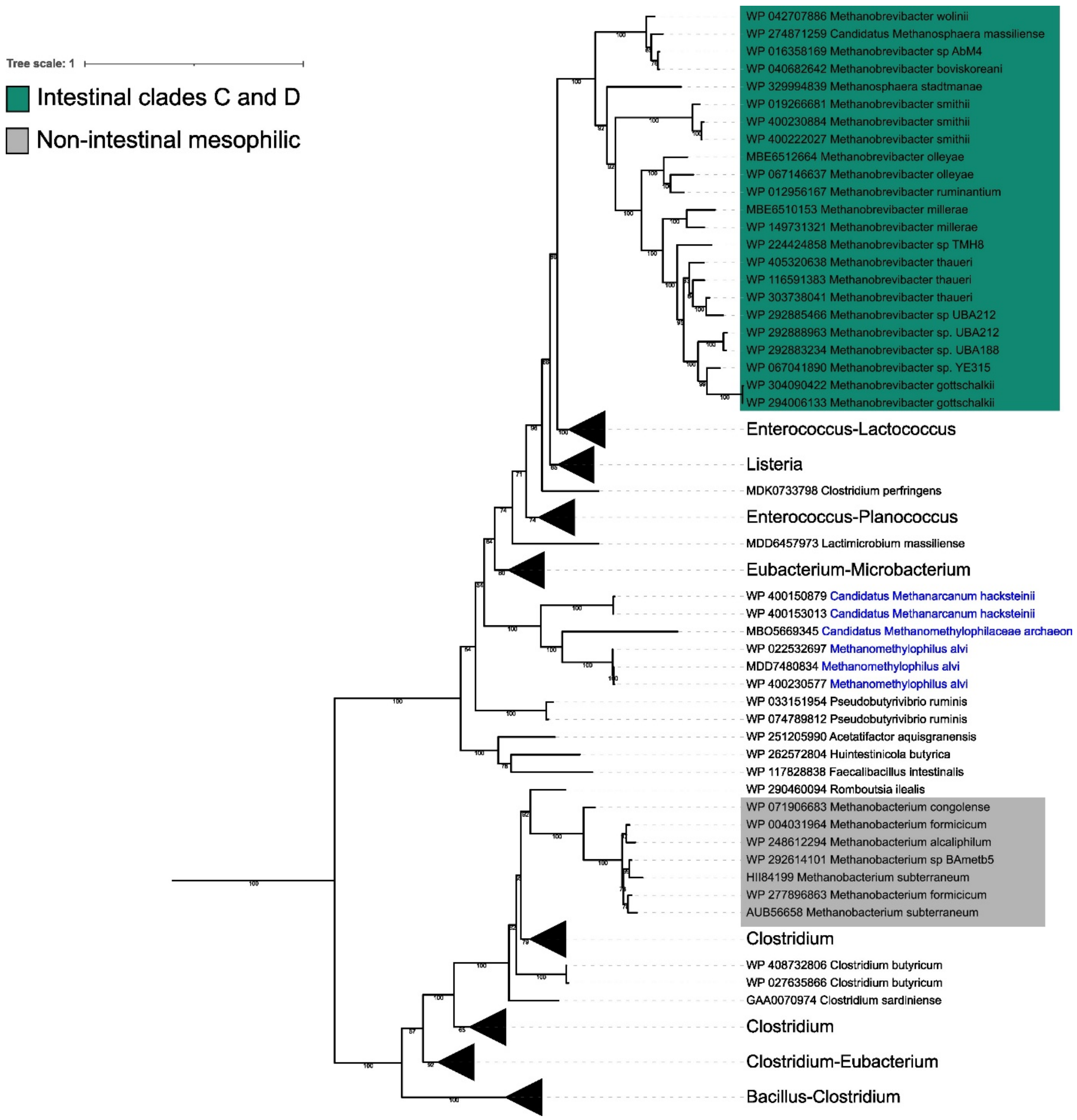

Figure S9. Phylogenetic tree of the bile hydrolases of *Methanobacteriota*. The maximum-likelihood tree was generated using IQ-TREE under the LG+F+I+G4 model of evolution. Values at the nodes indicate UFBoot support (1000 replicates). The scale bar indicates the number of substitutions per site.

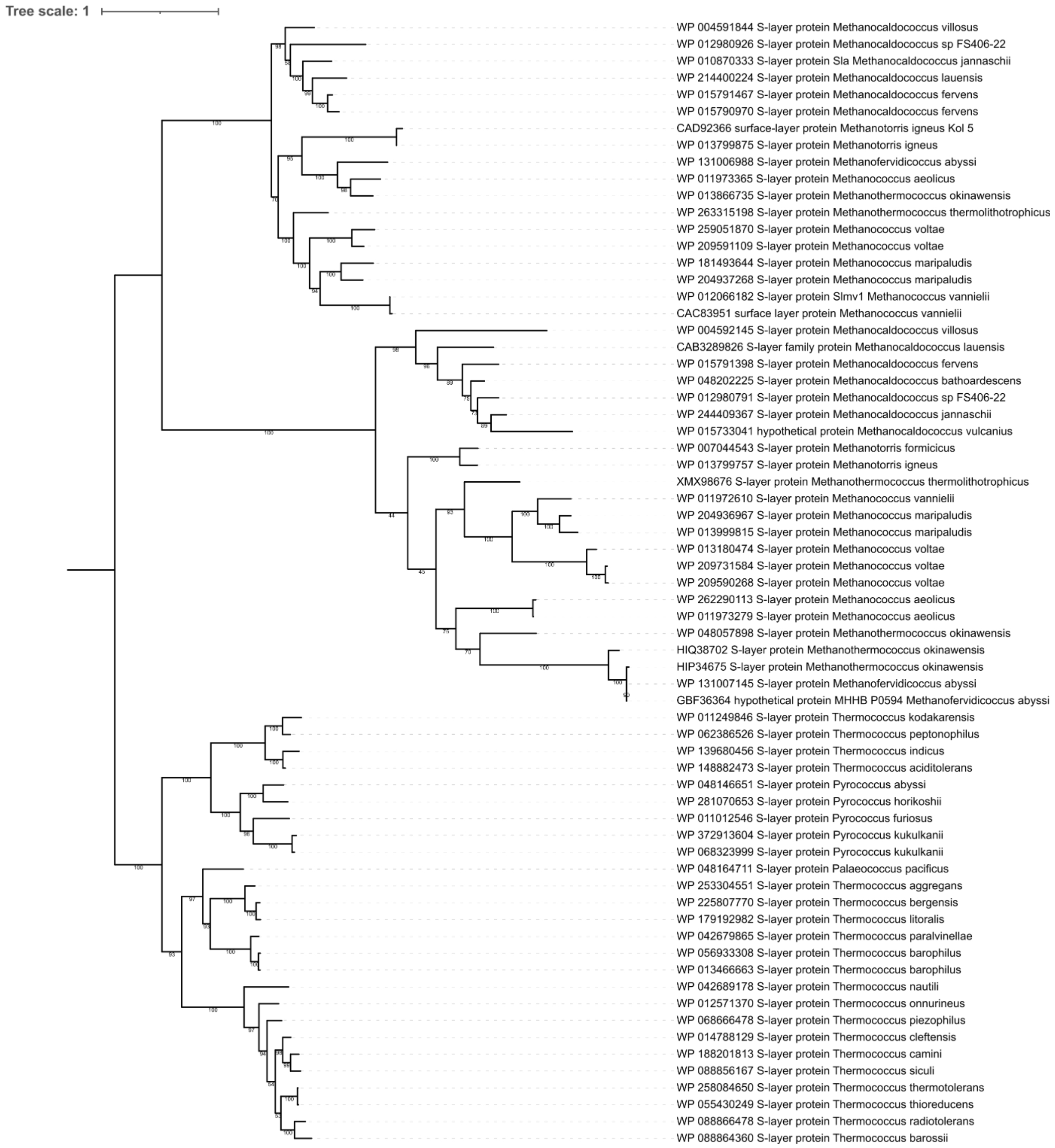

Figure S10. Phylogenetic tree of the S-layer proteins of *Methanobacteriota*. The maximum-likelihood tree was generated using IQ-TREE under the VT+F+I+G4 model of evolution. Values at the nodes indicate UFBoot support (1000 replicates). The scale bar indicates the number of substitutions per site.

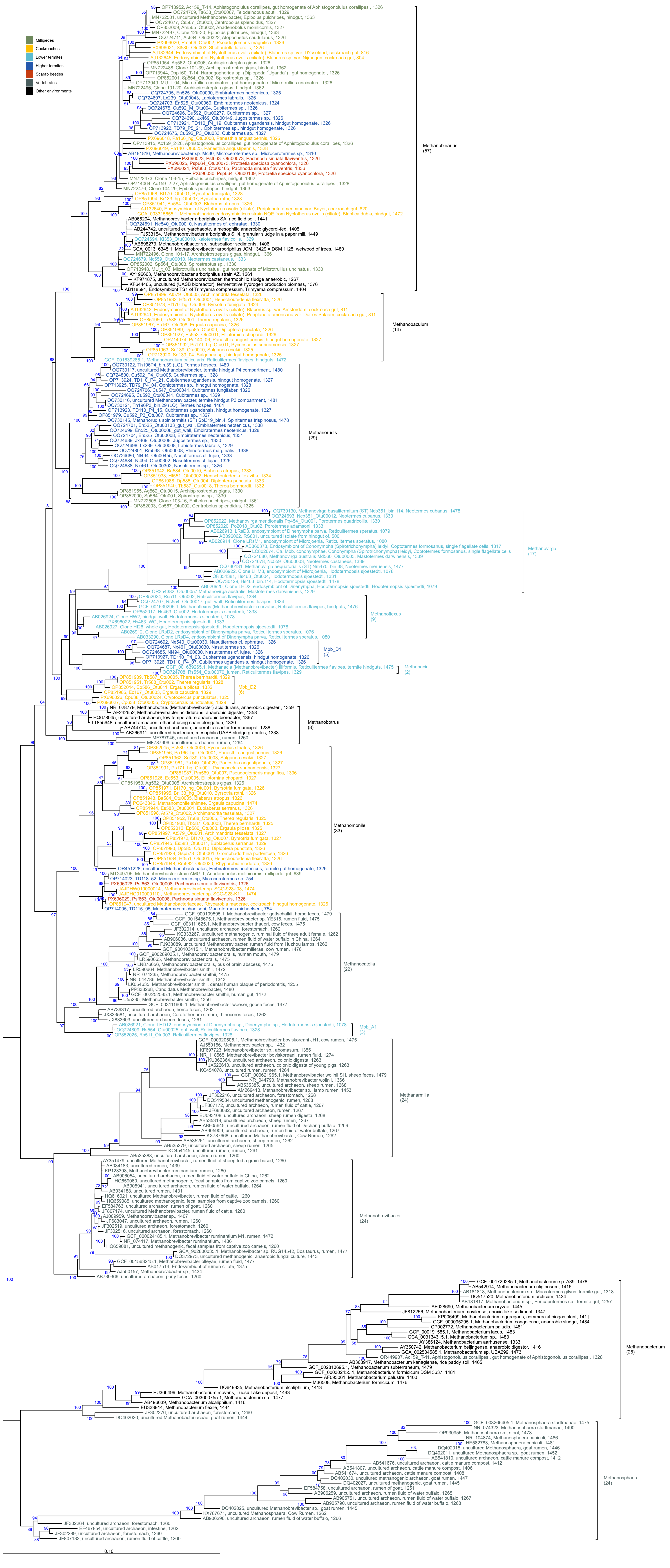

Figure S11. Phylogenetic 16S rRNA gene tree illustrating the relationship between various lineages of "*Methanobrevibacteraceae*". This is an expanded version of the tree in Fig. 5, showing accession numbers and isolation sources for sequences. Putative endosymbionts are shown in bold.
